# Comparison of full-dose gemcitabine/cisplatin, dose-reduced gemcitabine/cisplatin, and gemcitabine/carboplatin in real-world patients with advanced urothelial carcinoma

**DOI:** 10.1186/s12894-022-01139-9

**Published:** 2022-11-09

**Authors:** Kazuma Sugimoto, Satoru Taguchi, Kenjiro Kishitani, Taketo Kawai, Kazuki Masuda, Yu Nakamura, Manami Kinjo, Mitsuhiro Tambo, Jimpei Miyakawa, Yoshiyuki Akiyama, Yuta Yamada, Yusuke Sato, Daisuke Yamada, Tohru Nakagawa, Hiroshi Fukuhara, Haruki Kume

**Affiliations:** 1 Department of Urology, Kyorin University School of Medicine, 6-20-2 Shinkawa, Mitaka, Tokyo, 181-8611 Japan; 2 Department of Urology, Graduate School of Medicine, The University of Tokyo, 7-3-1 Hongo, Bunkyo-ku, Tokyo, 113-8655 Japan; 3 Department of Urology, Teikyo University School of Medicine, 2-11-1 Kaga, Itabashi-ku, Tokyo, 173-8605 Japan

**Keywords:** Advanced, Carboplatin, Cisplatin, Metastatic, Renal function, Urothelial carcinoma

## Abstract

**Background:**

While gemcitabine/cisplatin (GC) is the gold standard regimen for patients with advanced urothelial carcinoma (aUC), either dose-reduced GC or gemcitabine/carboplatin (GCa) is an alternative option for “cisplatin-unfit” patients. However, few studies have compared outcomes with these commonly used regimens in the real-world setting.

**Methods:**

We retrospectively reviewed patients with aUC who received full-dose GC, dose-reduced GC, or GCa as first-line salvage chemotherapy at two university hospitals between 2016 and 2020. Progression-free survival, cancer-specific survival, and overall survival, as well as best overall response and adverse event profiles, were compared among these three regimens.

**Results:**

Of 105 patients, 41, 27, and 37 patients received full-dose GC, dose-reduced GC, and GCa, respectively. Significant differences were noted in the patients’ baseline age, primary site, and renal function among the three regimens. Sixty-nine (65.7%) patients died during a median follow-up period of 14 months. There was no significant difference among the three regimens for all survival outcomes and best overall response. However, the complete response rate of dose-reduced GC (2/27, 7.4%) appeared inferior to that of full-dose GC (9/41, 22.0%) or GCa (6/37, 16.2%). Regarding adverse event profiles, no significant difference was observed among the three regimens, except for significantly fewer cases with elevated alanine aminotransferase in the GCa group compared with the other groups.

**Conclusions:**

This study compared the oncological and toxicological outcomes of full-dose GC, dose-reduced GC, and GCa in real-world patients with aUC. Unlike in the clinical trial setting, there were almost no significant differences among the three regimens.

## Background

For the last three decades, platinum-based chemotherapy has played a key role in the treatment of advanced urothelial carcinoma (aUC). Gemcitabine/cisplatin (GC) is currently the gold standard regimen for patients with aUC who are fit for cisplatin [1, 2], as well as dose-dense methotrexate/vinblastine/doxorubicin/cisplatin (ddMVAC) [3, 4]. However, 30–50% of patients with aUC are ineligible to receive cisplatin because of poor performance status, impaired renal function (creatinine clearance < 60 mL/min), hearing loss, peripheral neuropathy, and heart failure [5, 6]. For such “cisplatin-unfit” patients, gemcitabine/carboplatin (GCa), in which cisplatin in the GC regimen is replaced with another platinum-containing drug, carboplatin, can be an alternative [3, 4]. However, the efficacy of GCa was proven inferior to that of GC in a previous clinical trial [7]. The GC regimen with a reduced dosage of cisplatin (i.e., “dose-reduced GC”) can also be used for patients who are considered ineligible for “full-dose GC” for several reasons (typically, impaired renal function) in the real-world setting [8, 9]. However, few studies have compared the outcomes of these commonly used regimens (dose-reduced GC and GCa) [9]. Therefore, the present study aimed to investigate the oncological and toxicological outcomes of full-dose GC, dose-reduced GC, and GCa in real-world patients with aUC.

## Patients and methods

### Ethical approval and informed consent

The present study was conducted in accordance with the 1964 Declaration of Helsinki and its later amendments or comparable ethical standards. The study was approved by the Institutional Review Board (IRB) of the Graduate School of Medicine and Faculty of Medicine, The University of Tokyo (approval number: 10565) and the IRB of Kyorin University School of Medicine (approval number: 1262). Given the retrospective nature of the study, the requirement for informed consent was waived by the IRB of the Graduate School of Medicine and Faculty of Medicine, The University of Tokyo and the IRB of Kyorin University School of Medicine.

### Study population

We retrospectively reviewed 107 patients with aUC who received full-dose GC, dose-reduced GC, or GCa as first-line salvage chemotherapy at either The University of Tokyo Hospital or Kyorin University Hospital between January 2016 and August 2020. We excluded two patients owing to the lack of appropriate image evaluation after starting chemotherapy, leaving 105 patients for inclusion in the final analysis (The University of Tokyo Hospital, *n* = 35; Kyorin University Hospital, *n* = 70).

### Treatment and assessment

The regimen choice for each patient (full-dose GC, dose-reduced GC, or GCa) was dependent on the attending physician’s decision. Generally, full-dose GC was used for patients with good performance status and normal renal function, while either dose-reduced GC or GCa was selected for those with poor performance status and/or impaired renal function. The full-dose GC regimen consisting of 1000 mg/m^2^ gemcitabine on days 1, 8, and 15 and 70 mg/m^2^ cisplatin on day 2 was given every 28 days. The cisplatin dosage in the dose-reduced GC regimen was determined at the physician’s discretion and ranged from 50 to 80%; however, most (22/27, 81%) patients received the 75% dosage. The GCa regimen consisting of 1000 mg/m^2^ gemcitabine on days 1 and 8 and area under the curve 5 carboplatin on day 1 was given every 21 days. All patients underwent evaluations every 1–3 months, which comprised routine blood tests and/or computed tomography. The treatment efficacy was assessed in accordance with the Response Evaluation Criteria in Solid Tumours (RECIST) v1.1 [10]. Adverse events were evaluated in accordance with the Common Terminology Criteria for Adverse events (CTCAE) v5.0 [11]. Estimated glomerular filtration rate (eGFR) was calculated using the revised formula for Japanese patients [12].

### Endpoints and follow-up

As oncological endpoints, progression-free survival (PFS), cancer-specific survival (CSS), overall survival (OS), and best overall response according to the RECIST v1.1 criteria [10] were compared among the three regimens. As toxicological endpoints, comprehensive adverse event profiles, namely abnormal laboratory data, rash, fatigue, and vomiting, were also assessed in accordance with the CTCAE v5.0 criteria [11], as stated above. Follow-up started on the day of initiating first-line chemotherapy. Follow-up information was obtained as of September 2021.

### Statistical analysis

Differences in patient characteristics among the three regimens were assessed using Student’s *t*-test or the χ^2^ test. Survival curves were generated using the Kaplan–Meier method, and the curves were compared using the log-rank test. A Cox proportional hazards regression model was used for univariate and multivariate analyses of PFS, CSS, and OS. All statistical analyses were performed using JMP Pro version 15.0.0 (SAS Institute, Cary, NC, USA), and *P* < 0.05 indicated a significant difference.

## Results

### Patient characteristics

The patients’ characteristics at the start of first-line chemotherapy are summarized in Table 1. Of the 105 patients, 41, 27, and 37 patients received full-dose GC, dose-reduced GC, and GCa, respectively. Patients treated with full-dose GC were younger, had less frequent upper urinary tract disease, and better renal function compared with the other groups. Fifteen of 41 (36.6%) patients in the full-dose GC group had a primary tumor in upper urinary tract or both, whereas 17 of 27 (63.0%) and 29 of 37 (78.4%) patients in the dose-reduced GC and GCa groups did so, respectively (*P* = 0.0040). Median eGFRs (mL/min/1.73 m^2^) were 68.1, 45.2, and 39.3, in the full-dose GC, dose-reduced GC, and GCa groups, respectively (*P* < 0.0001; note: two patients with missing data were excluded in the calculation of eGFR). There was no significant difference in the other parameters, namely Eastern Cooperative Oncology Group Performance Status (ECOG PS), resection of the primary site, prior neoadjuvant/adjuvant chemotherapy, and metastatic sites, among the three regimens. The median number of chemotherapy cycles was 3 (interquartile range [IQR], 2–4), 3 (IQR, 2–4), and 3 (IQR, 1.5–6.5), with full-dose GC, dose-reduced GC, and GCa, respectively (*P* = 0.26). During the study period after first-line chemotherapy, 25 (23.8%) patients received pembrolizumab as later-line treatment (full-dose GC, *n* = 11; dose-reduced GC, *n* = 9; GCa, *n* = 5).

**Table 1:** Patient characteristics (*n* = 105)

Parameter	Total (*n* = 105)	Full-dose GC (*n* = 41)	Dose-reduced GC (*n* = 27)	GCa (*n* = 37)	*P* value
Age, years, median (IQR)	74 (69–79)	72 (64–77)	76 (69–80)	75 (71–80)	0.0076^a^*
Sex, no. (%)					0.55^b^
Male	80 (76.2)	29 (70.7)	22 (81.5)	29 (78.4)	
Female	25 (23.8)	12 (29.3)	5 (18.5)	8 (21.6)	
ECOG PS, no. (%)					0.43^b^
0	82 (68.6)	31 (75.6)	18 (66.7)	23 (62.2)	
≥ 1	33 (31.4)	10 (24.4)	9 (33.3)	14 (37.8)	
Primary site, no. (%)					0.0040^b^*
Bladder	44 (41.9)	26 (63.4)	10 (37.0)	8 (21.6)	
Upper urinary tract	46 (43.8)	13 (31.7)	12 (44.4)	21 (56.8)	
Both	15 (14.3)	2 (4.9)	5 (18.5)	8 (21.6)	
Serum creatinine, mg/dL, median (IQR)^†^	1.04 (0.75–1.38)	0.75 (0.67–1.02)	1.11 (0.91–1.39)	1.36 (0.99–1.55)	0.0012^a^*
eGFR, mL/min/1.73 m^2^, median (IQR)^†^	49.9 (39.8–67.3)	68.1 (54.8–84.3)	45.2 (39.3–61.2)	39.3 (34.0–48.6)	< 0.0001^a^*
Resection of primary site, no. (%)	52 (49.5)	19 (46.3)	10 (37.0)	23 (62.2)	0.12^b^
Prior neoadjuvant/adjuvant chemotherapy, no. (%)	11 (10.5)	4 (9.8)	2 (7.4)	5 (13.5)	0.72^b^
Lymph node metastasis, no. (%)	70 (66.7)	25 (61.0)	23 (85.2)	22 (59.5)	0.060^b^
Visceral metastasis, no. (%)	56 (53.3)	23 (56.1)	12 (44.4)	21 (56.8)	0.56^b^
Lung metastasis, no. (%)	37 (35.2)	18 (43.9)	8 (29.6)	11 (29.7)	0.33^b^
Bone metastasis, no. (%)	14 (13.3)	5 (12.2)	2 (7.41)	7 (18.9)	0.39^b^
Liver metastasis, no. (%)	13 (12.4)	2 (4.9)	3 (11.1)	8 (21.6)	0.79^b^
Follow–up duration, months, median (IQR)	14 (7–24)	14 (5–28.5)	15 (8–24)	11 (6–23)	0.76^a^

*ECOG PS* Eastern Cooperative Oncology Group Performance Status, *eGFR* estimated glomerular filtration rate, *GC* gemcitabine/cisplatin, *GCa* gemcitabine/carboplatin, *IQR* interquartile range

*Statistically significant, ^†^excluding two patients with missing data, ^a^Student’s *t*-test, ^b^χ^2^ test

### Oncological outcomes

**Fig. 1:**
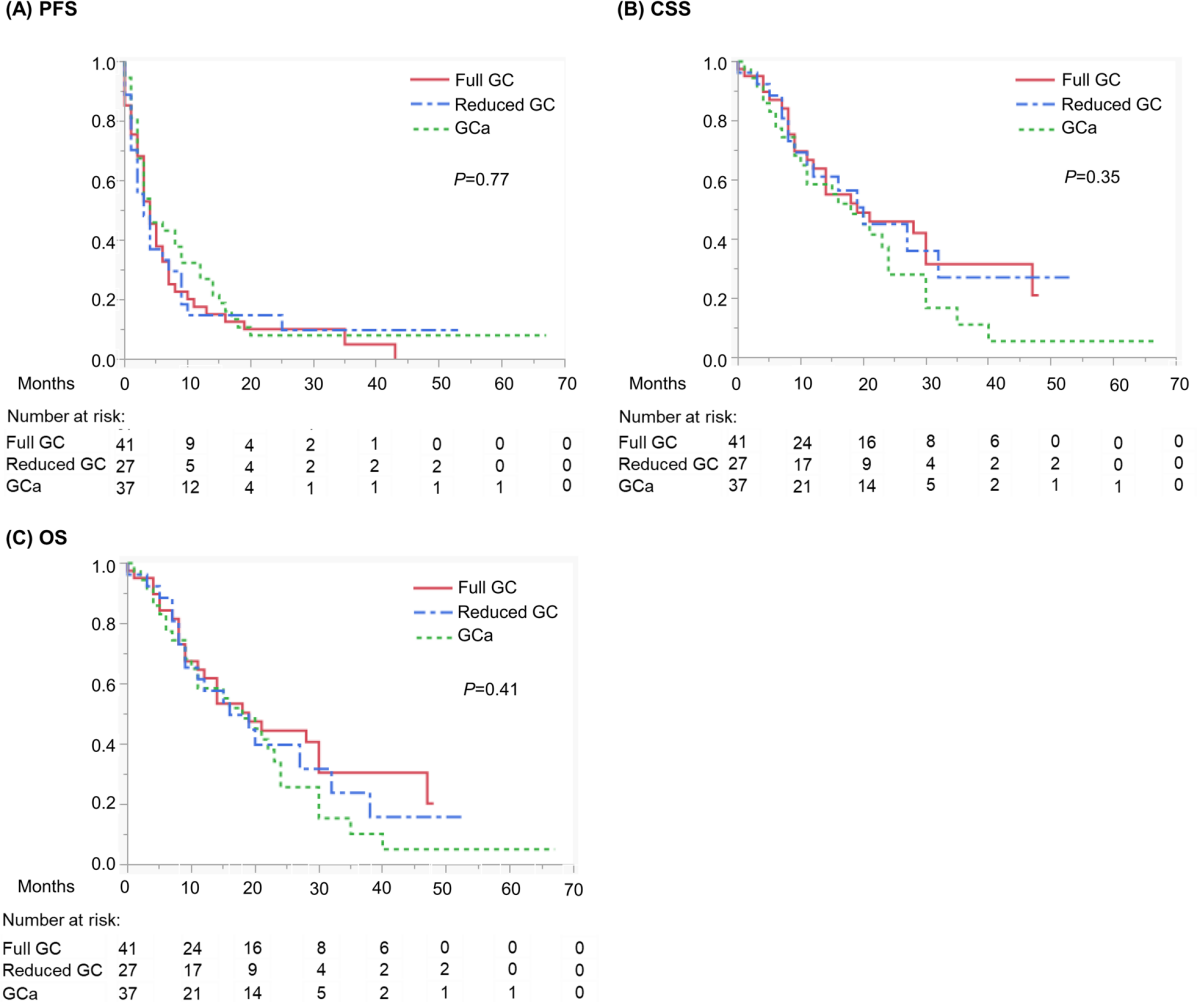
Kaplan–Meier curves depicting **A** PFS, **B** CSS, and **C** OS of patients with aUC treated with full-dose GC, dose-reduced GC, or GCa. Abbreviations: *aUC* Advanced urothelial carcinoma, *CSS* Cancer-specific survival, *GC* Gemcitabine/cisplatin, *GCa* Gemcitabine/carboplatin, *OS* Overall survival, *PFS* Progression-free survival

The median follow-up and survival times were 14 (IQR, 7–24) months and 18 (IQR, 8–32) months, respectively. Overall, 91 (86.7%) patients experienced disease progression, 64 (61.0%) died of aUC, and 5 (4.8%) died from other causes (pneumonia, *n* = 2; congestive heart failure, *n* = 1; myocarditis, *n* = 1; and gastric cancer, *n* = 1). There were no significant differences in PFS, CSS, and OS among the three regimens (Fig. 1).

Univariate analyses of PFS associated ECOG PS and resection of the primary site with the outcome, and multivariate analysis identified no resection of the primary site as an independent predictor of shorter PFS (Table 2). Univariate analyses of CSS associated ECOG PS, resection of the primary site, lymph node metastasis, and liver metastasis with the outcome, and multivariate analysis revealed ECOG PS ≥ 1, lymph node metastasis, and liver metastasis as independent predictors of shorter CSS (Table 3). Univariate and multivariate analyses of OS identified the same prognosticators as those identified for CSS (Table 4).

**Table 2:** Univariate and multivariate Cox proportional hazards regression analyses of PFS

Parameter	Cutoff	Univariate		Multivariate	
		HR (95% CI)	*P*	HR (95% CI)	*P*
Age (years)	Continuous	1.00 (0.98–1.02) per score	0.98		
Sex	Male	Reference	0.80		
	Female	0.94 (0.56–1.50)			
ECOG PS	0	Reference	0.043*	Reference	0.13
	≥ 1	1.57 (1.00–2.39)		1.41 (0.90–2.19)	
eGFR (mL/min/1.73 m^2^)	< 60	Reference	0.15		
	≥ 60	1.36 (0.89–2.06)			
Primary site	Bladder	Reference	0.52		
	Upper urinary tract	0.84 (0.54–1.31)			
	Both	1.18 (0.63–2.08)			
Resection of primary site	No	Reference	0.018*	Reference	0.047*
	Yes	0.61 (0.41–0.92)		0.66 (0.43–0.99)	
Prior neoadjuvant/adjuvant chemotherapy	No	Reference	0.66		
	Yes	1.15 (0.58–2.08)			
Lymph node metastasis	No	Reference	0.31		
	Yes	1.24 (0.82–1.92)			
Lung metastasis	No	Reference	0.68		
	Yes	1.09 (0.71–1.64)			
Bone metastasis	No	Reference	0.48		
	Yes	0.80 (0.41–1.42)			
Liver metastasis	No	Reference	0.22		
	Yes	1.45 (0.77–2.51)			
First-line regimen	Full-dose GC	Reference	0.80		
	Dose-reduced GC	0.95 (0.56–1.57)			
	GCa	0.85 (0.53–1.36)			

*CI* confidence interval, *ECOG PS* Eastern Cooperative Oncology Group Performance Status, *eGFR* estimated glomerular filtration rate, *GC* gemcitabine/cisplatin, *GCa* gemcitabine/carboplatin, *HR* hazard ratio, *PFS* progression-free survival

*Statistically significant

**Table 3:** Univariate and multivariate Cox proportional hazards regression analyses of CSS

Parameter	Cutoff	Univariate		Multivariate	
		HR (95% CI)	*P*	HR (95% CI)	*P*
Age (years)	Continuous	1.01 (0.99–1.04) per score	0.31		
Sex	Male	Reference	0.62		
	Female	0.86 (0.47–1.53)			
ECOG PS	0	Reference	0.019*	Reference	0.013*
	≥ 1	1.89 (1.09–3.17)		2.04 (1.14–3.56)	
eGFR (mL/min/1.73 m^2^)	< 60	Reference	0.57		
	≥ 60	1.16 (0.68–1.93)			
Primary site	Bladder	Reference			
	Upper urinary tract	1.12 (0.65–1.96)			
	Both	1.70 (0.79–3.42)			
Resection of primary site	No	Reference	0.0039*	Reference	0.067
	Yes	0.48 (0.29–0.79)		0.61 (0.36–1.03)	
Prior neoadjuvant/adjuvant chemotherapy	No	Reference	0.47		
	Yes	1.30 (0.60–2.51)			
Lymph node metastasis	No	Reference	0.020*	Reference	0.0019*
	Yes	1.91 (1.13–3.39)		2.51 (1.43–4.58)	
Lung metastasis	No	Reference	0.62		
	Yes	0.88 (0.51–1.46)			
Bone metastasis	No	Reference	0.79		
	Yes	1.10 (0.52–2.07)			
Liver metastasis	No	Reference	0.021*	Reference	0.0045*
	Yes	2.16 (1.07–3.99)		2.71 (1.30–5.23)	
First-line regimens	Full-dose GC	Reference	0.37		
	Dose-reduced GC	1.03 (0.52–1.95)			
	GCa	1.45 (0.82–2.56)			

*CI* confidence interval, *CSS* cancer-specific survival, *ECOG PS* Eastern Cooperative Oncology Group Performance Status, *eGFR* estimated glomerular filtration rate, *GC* gemcitabine/cisplatin, *GCa* gemcitabine/carboplatin, *HR* hazard ratio

*Statistically significant

**Table 4:** Univariate and multivariate Cox proportional hazards regression analyses of OS

Parameter	Cutoff	Univariate		Multivariate	
		HR (95% CI)	*P*	HR (95% CI)	*P*
Age (years)	Continuous	1.02 (0.99–1.04) per score	0.19		
Sex	Male	Reference	0.43		
	Female	0.78 (0.41–1.39)			
ECOG PS	0	Reference	0.011*	Reference	0.0074*
	≥ 1	1.93 (1.15–3.18)		2.09 (1.20–3.57)	
eGFR (mL/min/1.73 m^2^)	< 60	Reference	0.52		
	≥ 60	1.17 (0.71–1.92)			
Primary site	Bladder	Reference	0.17		
	Upper urinary tract	1.06 (0.63–1.82)			
	Both	1.85 (0.91–3.57)			
Resection of primary site	No	Reference	0.0043*	Reference	0.079
	Yes	0.49 (0.30–0.80)		0.64 (0.38–1.05)	
Prior neoadjuvant/adjuvant chemotherapy	No	Reference	0.61		
	Yes	1.20 (0.55–2.31)			
Lymph node metastasis	No	Reference	0.038*	Reference	0.0032*
	Yes	1.73 (1.05–2.95)		2.28 (1.34–4.01)	
Lung metastasis	No	Reference	0.62		
	Yes	0.88 (0.53–1.44)			
Bone metastasis	No	Reference	0.71		
	Yes	1.13 (0.56–2.84)			
Liver metastasis	No	Reference	0.014*	Reference	0.0029*
	Yes	2.20 (1.12–3.96)		2.72 (1.35–5.09)	
First-line regimens	Full-dose GC	Reference	0.73		
	Dose-reduced GC	1.11 (0.59–2.03)			
	GCa	1.25 (0.72–2.18)			

*CI* confidence interval, *ECOG PS* Eastern Cooperative Oncology Group Performance Status, *eGFR* estimated glomerular filtration rate, *GC* gemcitabine/cisplatin, *GCa* gemcitabine/carboplatin, *HR* hazard ratio, *OS* overall survival

*Statistically significant

**Fig. 2:**
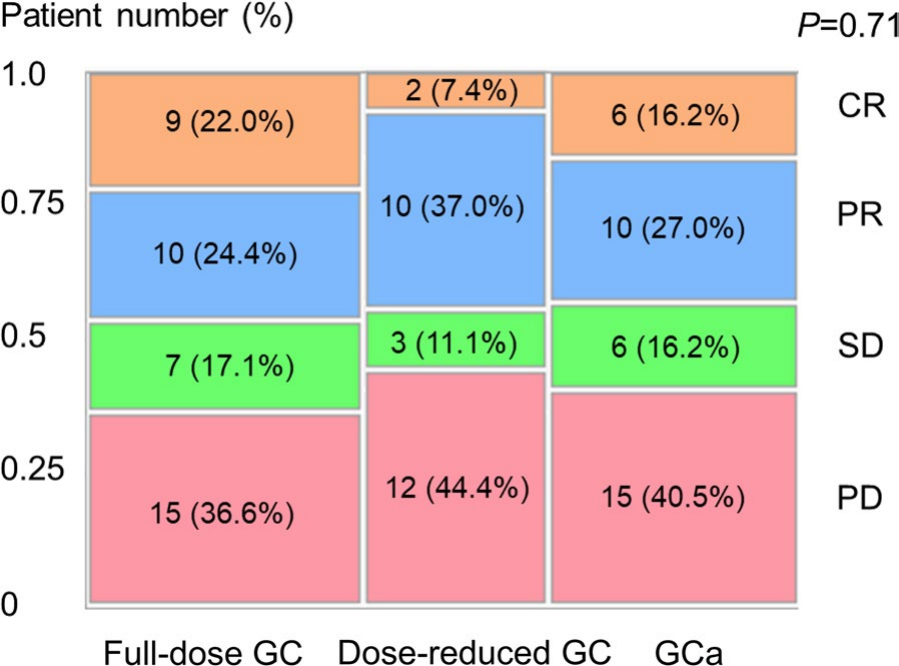
Best overall response results of the three regimens (full-dose GC, dose-reduced GC, and GCa). Abbreviations: *CR* complete response, *GC* gemcitabine/cisplatin, *GCa* gemcitabine/carboplatin, *PD* progressive disease, *PR* partial response, *SD* stable disease

Figure 2 shows the best overall response results of the three regimens. Overall, 47 (44.8%) patients achieved an objective response (complete response [CR] + partial response [PR]). There were no significant differences in the objective response rate, durable response rate (CR + PR + stable disease), or progressive disease rate among the three regimens. Notably, the CR rate of dose-reduced GC (2/27, 7.4%) appeared inferior to that of full-dose GC (9/41, 22.0%) or GCa (6/37, 16.2%), although there was no significant difference (*P* = 0.28).

### Toxicological outcomes

**Fig. 3:**
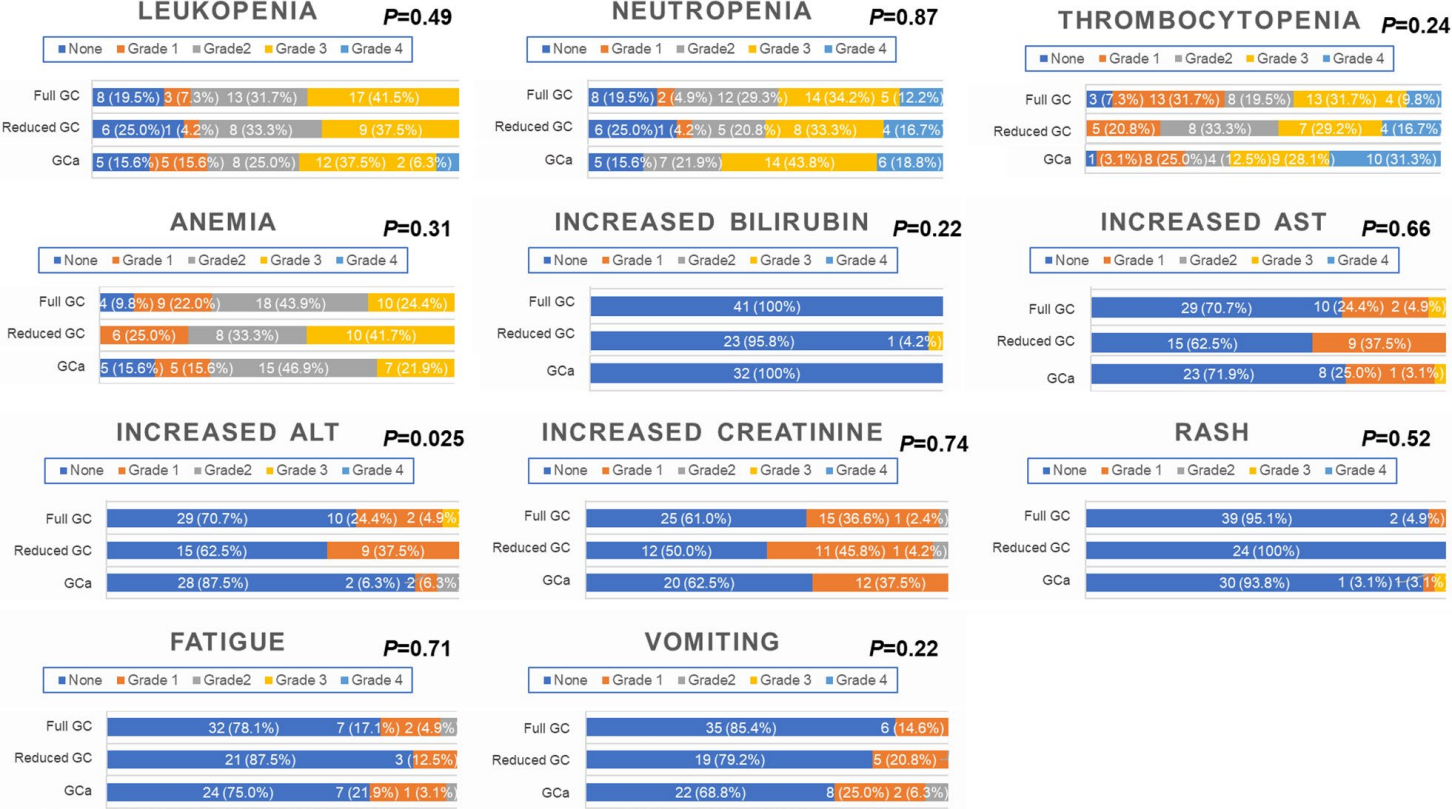
Comprehensive adverse event profiles among the three regimens (full-dose GC, dose-reduced GC, and GCa). The analysis was conducted for 97 patients with sufficient data (GCa, *n* = 32; full-dose GC, *n* = 41; dose-reduced GC, *n* = 24). Abbreviations: *ALT* alanine aminotransferase, *AST* aspartate aminotransferase, *GC* gemcitabine/cisplatin, *GCa* gemcitabine/carboplatin

Figure 3 shows the comprehensive adverse event profiles among the three regimens, which were evaluated in 97 patients with sufficient data (full-dose GC, *n* = 41; dose-reduced GC, *n* = 24; GCa, *n* = 32). There were no significant differences in the rates of hematological toxicities (leukopenia, neutropenia, thrombocytopenia, and anemia), most hepatic toxicities (increased bilirubin and aspartate aminotransferase), renal toxicity (increased creatinine), rash, fatigue, and vomiting, among the three regimens. There were significantly fewer cases with increased alanine aminotransferase in the GCa group compared with the other groups (*P* = 0.025).

## Discussion

The present study compared the oncological and toxicological outcomes of full-dose GC, dose-reduced GC, and GCa in real-world patients with aUC. Unlike in the clinical trial setting, there were almost no significant differences in all endpoints assessed among the three regimens. Specifically, no significant difference was observed for all survival outcomes (PFS, CSS, and OS) and best overall response among the three regimens. Furthermore, the CR rate of dose-reduced GC (2/27, 7.4%) appeared inferior to that of full-dose GC (9/41, 22.0%) or GCa (6/37, 16.2%), albeit without a significant difference (*P* = 0.28). Similarly, no significant difference was observed for all but one toxicological endpoint among the three regimens, whereas there were significantly fewer cases with elevated alanine aminotransferase in patients undergoing GCa compared with the concentration in those receiving the other regimens (*P* = 0.025).

A randomized phase 2 trial comparing GC and GCa in “cisplatin-fit” patients (creatinine clearance ≥ 60 mL/min) reported an overall response rate of 49.1% for GC (CR: 14.5%; PR: 34.5%) and 40.0% for GCa (CR: 1.8%; PR: 38.2%). The authors also reported that median OS was 12.8 months and 9.8 months for GC and GCa, respectively, while no differences between the regimens were noted for the overall toxicity profiles [7]. Therefore, current guidelines do not recommend the use of GCa for “cisplatin-fit” patients with aUC [3, 4]. In contrast, another phase 2/3 trial comparing GCa and methotrexate/carboplatin/vinblastine (M-CAVI) in “cisplatin-unfit” patients (creatinine clearance 30–60 mL/min and/or ECOG PS 2) reported that the best overall response rates were 41.2% for GCa versus 30.3% for M-CAVI (*P* = 0.08) and that median OS was 9.3 months and 8.1 months for GCa and M-CAVI, respectively (*P* = 0.64). However, severe acute toxicity was observed in 9.3% of the patients receiving GCa and 21.2% of those receiving M-CAVI [13, 14]. Therefore, current guidelines recommend the use of GCa for “cisplatin-unfit” patients with aUC [3, 4]. Accordingly, the current European Association of Urology guideline classifies patients into the following three categories (with recommended first-line regimens): (1) fit for cisplatin (GC or ddMVAC is recommended); (2) unfit for cisplatin but fit for carboplatin (GCa is recommended); and (3) unfit for any platinum-based chemotherapy (immune checkpoint inhibitors, such as pembrolizumab and atezolizumab are considered) [3].

Our results may be inconsistent with those of the abovementioned clinical trials because the efficacy of GCa was comparable to that of GC, in our study. This difference is probably because of the retrospective design, small sample size, and selection bias. Nevertheless, given that GCa might have a higher CR rate than that of dose-reduced GC and that toxicities associated with GCa were similar (or lower) than those associated with dose-reduced GC, GCa might be preferable for “cisplatin-unfit” patients, rather than dose-reduced GC. The use of GCa could avoid toxicities induced by the long-term use of cisplatin, such as neurotoxicity, ototoxicity, and nephrotoxicity [15]. For reference, Ichioka et al. previously reported that the oncological outcomes of dose-reduced GC were inferior to those of full-dose GC in patients with eGFR < 60 mL/min/1.73 m^2^ [8]. More recently, Miyake et al. compared first-line full-dose GC, dose-reduced GC, and GCa in terms of the response to subsequent pembrolizumab treatment [9]. The authors reported that the response to pembrolizumab after dose-reduced GC was inferior to that after GCa in cisplatin-unfit patients with aUC and, thus, concluded that dose-reduced GC is not recommended for such patients [9].

Regarding the prognostic factors, poor performance status (ECOG PS ≥ 1) and liver metastasis were shown to be independent predictors of shorter CSS and OS (Tables 3, 4), in this study. These two factors have been reported as critical prognostic factors for patients with aUC [16–18], even in the era of immune checkpoint inhibitors [18]. Our results were similar to findings in the previous literature. In contrast, the type of first-line regimen (full-dose GC, dose-reduced GC, or GCa) was not associated with all of the survival endpoints that we assessed (PFS, CSS, and OS) even in the univariate analysis (Tables 2, 3, 4).

Although not assessed in the present study, gemcitabine plus split‑dose cisplatin (“GC split”) has been another well-known option for cisplatin-unfit patients with aUC [3, 19–23]. Several small-scale studies have evaluated “GC split” in UC patients with impaired renal function (creatinine clearance: 40–60 ml/min) using different split-dose schedules, all of which reported its feasibility and potential efficacy [19–23]. Nevertheless, given the study design of these previous studies (a small phase I/II trial or retrospective study), prospective randomized trials comparing “GC split” with conventional GC are warranted to validate this modified regimen [3].

The limitations of this study are the retrospective design, small sample size, and selection bias, as stated above. Additionally, owing to the multi-institutional retrospective study design, the dose reduction rate of cisplatin was not uniform. Nevertheless, this study might add additional evidence of the real-world outcomes of the commonly used regimens in patients eligible for platinum-based chemotherapy.

## Conclusions

In real-world patients with aUC, there were almost no significant differences in both oncological and toxicological outcomes among patients receiving full-dose GC, dose-reduced GC, and GCa, unlike findings in the clinical trial setting.

## Data Availability

Because of ethical restrictions, the raw data underlying this study are available from the corresponding author upon reasonable request.

## Abbreviations

aUC: Advanced urothelial carcinoma
CR: Complete response
CSS: Cancer-specific survival
CTCAE: Common Terminology Criteria for Adverse events
ddMVAC: Dose-dense methotrexate/vinblastine/doxorubicin/cisplatin
ECOG PS: Eastern Cooperative Oncology Group Performance Status
eGFR: Estimated glomerular filtration rate
GC: Gemcitabine/cisplatin
GCa: Gemcitabine/carboplatin
IQR: Interquartile range
IRB: Institutional Review Board
M-CAVI: Methotrexate/carboplatin/vinblastine
OS: Overall survival
PFS: Progression-free survival
PR: Partial response
RECIST: Response evaluation criteria in solid tumours
